# Peripheral lymphocytes and lactate dehydrogenase correlate with response and survival in head and neck cancers treated with immune checkpoint inhibitors

**DOI:** 10.1002/cam4.5697

**Published:** 2023-02-21

**Authors:** Cassie Pan, Qian Vicky Wu, Jenna Voutsinas, Jeffrey J. Houlton, Brittany Barber, Zain H. Rizvi, Emily Marchiano, Neal Futran, George E. Laramore, Jay J. Liao, Upendra Parvathaneni, Renato G. Martins, Jonathan R. Fromm, Cristina P. Rodriguez

**Affiliations:** ^1^ Department of Otolaryngology‐Head and Neck Surgery University of Washington Seattle Washington USA; ^2^ Clinical Research Division Fred Hutchinson Cancer Center Seattle Washington USA; ^3^ Head & Neck Specialists Charleston South Carolina; ^4^ Department of Radiation Oncology University of Washington Seattle Washington USA; ^5^ Division of Hematology, Oncology and Palliative Care Department of Medicine Virginia Commonwealth University Richmond Virginia USA; ^6^ Department of Laboratory Medicine and Pathology University of Washington Seattle Washington USA; ^7^ Division of Oncology, Department of Medicine University of Washington Seattle Washington USA

**Keywords:** biomarkers, check point control, head and neck cancer, medical oncology, prognostic factor

## Abstract

**Background:**

Little is known regarding associations between peripheral blood biomarkers (PBBMs) and survival, response, and toxicity in recurrent/metastatic head and neck squamous cell carcinomas (R/M HNSCC) treated with immune checkpoint inhibitors (ICIs).

**Methods:**

In this single‐institution retrospective cohort study, a dataset of patients with R/M HNSCC treated with ICIs between 08/2012–03/2021 was established, including demographic and clinicopathologic characteristics. Pretreatment PBBMs were collected and evaluated for associations with grade ≥3 adverse events (G ≥ 3AE) by CTCAEv5, objective response (ORR) by RECIST 1.1, overall survival (OS), and progression‐free survival (PFS). Multivariable models for each outcome were created using elastic net variable selection.

**Results:**

Our study included 186 patients, with 51 (27%) demonstrating complete or partial response to immunotherapy. Multivariable models adjusted for ECOG performance status (PS), p16, and smoking demonstrated that pretreatment higher LDH and absolute neutrophils, as well as lower percent lymphocytes correlated with worse OS and PFS. Higher LDH and lower % lymphocytes also correlated with worse ORR.

**Conclusions:**

In the largest study to date examining PBBMs in ICI‐treated R/M HNSCCs, our variable selection method revealed PBBMs prognostic for survival and response to immunotherapy. These biomarkers warrant further investigation in a prospective study along with validation with CPS biomarker.

## 1 INTRODUCTION

With the FDA approval of PD‐1 inhibitors for treatment of recurrent or metastatic (R/M) head and neck squamous cell carcinoma (HNSCC) in 2016, treatment options and survival have improved for this deadly disease.^1^ However, immune checkpoint inhibitors (ICIs) are not without drawbacks, as they are more expensive than traditional chemotherapy regimens, introduce risks of immune‐related adverse events, and lead to response in only about one‐third or less of patients.^1^, ^2^, ^3^, ^4^ Patient selection for pembrolizumab is currently guided by combined proportion score (CPS), a measure of PD‐L1 expression, which predicts benefit from these drugs. However, even in the biomarker‐enriched population of CPS ≥20, only a minority of patients respond to ICIs as demonstrated in the landmark KEYNOTE‐048 phase III clinical trial.^5^ Thus, improved biomarkers for survival, response, and toxicities to ICI treatment of R/M HNSCC are needed.

Peripheral blood biomarkers (PBBMs) have been studied in head and neck cancer patients, but little is known regarding their utility in the setting of treatment with ICIs. In a small pilot study of R/M HNSCC and salivary gland cancers treated on a clinical trial with pembrolizumab and vorinostat, a histone deacetylase inhibitor, we observed that higher pretreatment neutrophil‐to‐lymphocyte ratio (NLR) and neutrophils, as well as lower lymphocytes correlated with worse overall survival (OS) and progression‐free survival (PFS).^6^ We also found that higher NLR correlated with increased rates of serious toxicities. These results corroborate existing literature examining PBBMs in ICI‐treated HNSCC, which to this point consist of relatively small studies with limited follow‐up times.^7^, ^8^ In this study, we aimed to expand upon those results and explore associations of PBBMs with survival, response, and toxicity in patients with R/M HNSCC treated with ICIs with mature follow‐up times.

## 2 METHODS

### 2.1 Study design and population

This single‐institution retrospective cohort study included patients with R/M HNSCC treated with ICIs between August 2012 and March 2021 at the University of Washington Medical Center. Patients without pre‐treatment peripheral blood data were excluded. Follow‐up continued until February 15, 2022. Adverse events, as reported and graded by Common Terminology Criteria for Adverse Events version 5 (CTCAEv5) according to the treating physician, that occurred within the first 100 days of ICI treatment initiation were recorded. Objective response (ORR) was determined both radiographically by RECIST 1.1 criteria and clinically by the patient's primary oncologist, as formal RECIST assessments were not performed routinely outside of a clinical trial. Blinding, group randomization, and power analysis were not relevant to this study. This study was reviewed and approved by the Fred Hutchinson Cancer Research Institutional Review Board.

Demographics including age at initiation of ICI treatment, sex, and race were reported. Performance status (PS) was evaluated on the first day of ICI administration using the Eastern Cooperative Oncology Group (ECOG) scale as documented by the patient's oncologist. History of immunosuppression was defined as HIV/AIDS, chronic steroid use, hematologic malignancy, or autoimmune disease on immunosuppressive medications. Additional patient characteristics recorded included smoking history, alcohol use, p16 status, and CPS. Smoking history was defined as current smoker, former smoker (quit ≥12 months prior to treatment initiation), or never smoker. Alcohol use was categorized according to CDC guidelines, with heavy drinking defined as ≥8 drinks/week for women or ≥15 drinks/week for men, moderate drinking defined as ≤1 drink/day for women or ≤2 drinks/day for men, and none/rare defined as ≤1 drink per week. Data regarding prior lines of therapy was not available to be included in this study.

### 2.2 Correlative blood sample collection

Correlative peripheral blood samples within 24 h prior to starting ICI treatment were collected. The following peripheral blood parameters were evaluated: lactate dehydrogenase (LDH), platelets, neutrophils, lymphocytes, monocytes, eosinophils, neutrophil‐to‐lymphocyte ratio (NLR), and prognostic nutritional index (PNI). Percent (%) and absolute (abx) values for each cell type were examined. NLR was calculated as absolute neutrophil count divided by absolute lymphocyte count. PNI was calculated as defined in the literature as: 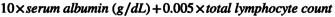 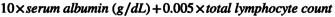.^9^, ^10^, ^11^, ^12^, ^13^

### 2.3 Statistical analysis

Summary statistics for continuous measures included sample size and median. Responses were summarized as frequencies and percentages. Cox regression was performed to explore associations with time‐to‐event outcomes, including overall survival (OS) and progression‐free survival (PFS). Logistic regression was performed for binary outcomes, including objective response (ORR) by RECIST 1.1 and grade ≥3 toxicities (G ≥ 3AE) by CTCAEv5 within 100 days of treatment initiation. Univariate analyses were conducted between each outcome and covariate of interest, adjusted for ECOG PS, p16 status, and smoking. The penalized regression Elastic Net was used for variable selection (using penalty factor = 0 for ECOG PS, p16, and smoking status in glmnet R package to allow those factors to always be included), and variables chosen were added to the final multivariable model. For statistical tests, a *p*‐value of <0.05 was considered statistically significant and 95% confidence intervals were reported.

## 3 RESULTS

### 3.1 Patient, tumor, and treatment characteristics

We identified 186 consecutive adult patients with R/M HNSCC treated with ICIs at our institution between August 2012 and March 2021. Patient and tumor characteristics, as well as treatments and responses are presented in Table 1. Median age was 64 (range 24–90) years and 145 (78%) were male. The majority 149 (82%) had ECOG PS of ≤1. Eighty‐one (44%) were never‐smokers and 60 (33%) had p16‐positive tumors. Most common primary sites included 73 oropharynx (39.2%), 49 oral cavity (26.3%), 14 nasopharynx (7.5%), and 14 larynx (7.5%). Other primary sites included sinonasal, salivary, cutaneous, auditory canal, hypopharynx, and unknown primary. The majority of patients were treated with pembrolizumab or nivolumab monotherapy (*n* = 140, 75.3%), with the remaining patients treated with an alternate PD‐1 inhibitor regimen, detailed in Table 1. Of the patients treated with an alternate PD‐1 inhibitor regimen, 25 (13.4%) received a combination of pembrolizumab and vorinostat, a histone deacetylase inhibitor, and five (2.7%) received cemiplimab (FDA‐approved for cutaneous SCC). One patient received a PD‐1 inhibitor along with chemotherapy, and no patients received a regimen containing radiation therapy. Fifty‐one (27%) demonstrated a complete or partial response to the ICI treatment regimen. Eight patients with immunosuppression were evaluated, including four with HIV, three with autoimmune disease on immunosuppressive medications, and one on chronic steroids. CPS score was only available in 33 (17%) of patients, as it was not routinely tested prior to 2019.

**TABLE 1:** Summary of patient and tumor characteristics, treatments, and responses.

	*N* = 186 (%)
Age	
Median (range)	64.0 (25.0–90.0)
Sex	
F	41 (22.0)
M	145 (78.0)
Race	
Asian	13 (7.0)
Black	7 (3.8)
Native American	3 (1.6)
Native Hawaiian/Pacific Islander	3 (1.6)
White	151 (81.2)
Declined/Unknown	9 (4.8)
Primary site	
Auditory canal	3 (1.6)
Cutaneous	7 (3.8)
Hypopharynx	7 (3.8)
Larynx	14 (7.5)
Nasopharynx	14 (7.5)
Oral cavity	49 (26.3)
Oropharynx	73 (39.2)
Parotid	1 (0.5)
Sinonasal	9 (4.8)
Unknown	9 (4.8)
ECOG PS	
Unknown	4
0	50 (27.5)
1	99 (54.4)
2	30 (16.5)
3	3 (1.6)
Smoking	
Current	26 (14.0)
Former	79 (42.5)
Never	81 (43.5)
Alcohol	
Unknown	5
Heavy	15 (8.3)
Moderate	59 (32.6)
None/rare	107 (59.1)
History of immunosuppression	
No	178 (95.7)
Yes	8 (4.3)
Charlson comorbidity index	
Median (range)	7.0 (2.0–13.0)
P16 status	
Unknown	2
Negative	124 (67.4)
Positive^b^	60 (32.6)
CPS score	
Unknown	153
Median (range)	31.0 (0.0–100.0)
Objective response	
Not evaluated	8 (4.3)
Complete response	14 (7.5)
Partial response	37 (19.9)
Stable disease	37 (19.9)
Progressive disease	90 (48.4)
Treatment	
Pembro or nivolumab monotherapy	140 (75.3)
Other PD‐1 regimen^a^	46 (24.7)

^a^ Other PD‐1 regimens included, in order of decreasing frequency: pembrolizumab + vorinostat (*n* = 25, 13.4%), cemiplimab (*n* = 5, 2.7%), durvalumab + AZD9150 (*n* = 3, 1.6%), pembrolizumab + tumor infiltrating lymphocytes (*n* = 2, 1.1%), pembrolizumab + carboplatin + paclitaxel (*n* = 2, 1.1%), nivolumab + ipilimumab (*n* = 2, 1.1%), nivolumab + anti‐IL21 (*n* = 2, 1.1%), durvalumab + MEDI068 (*n* = 2, 1.1%), pembrolizumab + epacadostat (*n* = 1, 0.5%), avelumab +4‐1BB agonist (*n* = 1, 0.5%), and avelumab +4‐1BB agonist + OX40 agonist (*n* = 1, 0.5%).

^b^ Of p16‐positive tumors, 51 (85.0%) were oropharyngeal. The remaining p16‐positive tumors originated from the larynx (*n* = 2, 3.3%), nasopharynx (*n* = 2, 3.3%), oral cavity (*n* = 2, 3.3%), and unknown primary (*n* = 3, 5.0%).

### 3.2 Univariate analyses of PBBMs associated with survival, toxicity, and response in HNSCC patients receiving ICIs

Baseline peripheral blood markers were evaluated in patients within 24 h prior to starting ICI treatment. Univariate analyses adjusted for ECOG PS, p16 status, and smoking revealed that higher LDH, % and abx neutrophils, abx monocytes, and NLR, and lower % and abx lymphocytes, % eosinophils, and PNI correlated with worse OS (Table 2). Elevated platelets, % and abx neutrophils, and NLR, and decreased % lymphocytes and PNI correlated with worse PFS (Table 3). No peripheral blood parameter reached significance for G ≥3AEs or ORR (Tables 4 and 5), although % lymphocytes, abx neutrophils, and LDH were borderline significant for ORR.

**TABLE 2:** Association of peripheral blood parameters with overall survival.

Baseline blood parameter^a^	Overall survival		
	HR	95% CI	*p*‐value
LDH	2.931	1.14–7.52	**0.025**
Platelets	2.530	0.78–8.24	0.123
Neutrophils (%)	1.040	1.02–1.06	**0.001**
Neutrophils (abx)	4.254	1.78–10.18	**0.001**
Lymphocytes (%)	0.162	0.08–0.33	**<0.001**
Lymphocytes (abx)	0.314	0.14–0.71	**0.005**
Monocytes (%)	0.854	0.28–2.58	0.780
Monocytes (abx)	2.575	1.03–6.43	**0.043**
Eosinophils (%)	0.687	0.48–0.99	**0.046**
Eosinophils (abx)	0.636	0.27–1.51	0.305
NLR	4.523	2.42–8.44	**<0.001**
PNI	0.940	0.90–0.98	**0.007**

^a^ All parameters are log10 transformed except neutrophils (%) and PNI.

Bold values are significant *p* values (*p* < 0.05).

**TABLE 3:** Association of peripheral blood parameters with progression‐free survival.

Baseline blood parameter^a^	Progression‐free survival		
	HR	95% CI	*p*‐value
LDH	2.068	0.85–5.05	0.111
Platelets	4.930	1.47–16.56	**0.010**
Neutrophils (%)	1.030	1.01–1.05	**0.003**
Neutrophils (abx)	4.956	2.12–11.59	**<0.001**
Lymphocytes (%)	0.218	0.11–0.45	**<0.001**
Lymphocytes (abx)	0.610	0.28–1.33	0.215
Monocytes (%)	0.387	0.13–1.15	0.088
Monocytes (abx)	2.235	0.90–5.57	0.084
Eosinophils (%)	0.701	0.49–1.01	0.055
Eosinophils (abx)	0.817	0.34–1.96	0.651
NLR	3.495	1.87–6.53	**<0.001**
PNI	0.940	0.90–0.99	**0.012**

^a^ All parameters are log10 transformed except neutrophils (%) and PNI.

Bold values are significant *p* values (*p* < 0.05).

**TABLE 4:** Association of peripheral blood parameters with grade ≥3 adverse events occurring within the first 100 days of starting ICI treatment.

Baseline blood parameter^a^	Grade ≥3 Adverse events		
	OR	95% CI	*p*‐value
LDH	1.720	0.31–9.58	0.536
Platelets	0.331	0.05–2.02	0.231
Neutrophils (%)	1.000	0.95–1.05	0.838
Neutrophils (abx)	0.758	0.14–4.12	0.749
Lymphocytes (%)	1.011	0.24–4.25	0.989
Lymphocytes (abx)	0.814	0.19–3.46	0.780
Monocytes (%)	2.874	0.33–24.81	0.337
Monocytes (abx)	1.568	0.29–8.37	0.598
Eosinophils (%)	1.513	0.72–3.19	0.277
Eosinophils (abx)	1.018	0.23–4.55	0.982
NLR	1.012	0.32–3.21	0.984
PNI	1.000	0.90–1.12	0.935

^a^ All parameters are log10 transformed except neutrophils (%) and PNI.

**TABLE 5:** Association of peripheral blood parameters with objective response rate.

Baseline blood parameter^a^	Objective response rate		
	OR	95% CI	*p*‐value
LDH	0.076	0.00–0.88	0.069
Platelets	0.410	0.03–5.84	0.498
Neutrophils (%)	0.980	0.94–1.02	0.267
Neutrophils (abx)	0.135	0.02–0.98	0.055
Lymphocytes (%)	5.730	0.95–40.51	0.066
Lymphocytes (abx)	1.199	0.20–6.85	0.839
Monocytes (%)	1.002	0.09–12.28	0.998
Monocytes (abx)	0.222	0.03–1.34	0.105
Eosinophils (%)	1.431	0.62–3.54	0.417
Eosinophils (abx)	0.940	0.15–5.38	0.946
NLR	0.297	0.07–1.25	0.101
PNI	1.070	0.97–1.18	0.175

^a^ All parameters are log10 transformed except neutrophils (%) and PNI.

### 3.3 Multivariable analysis of PBBMs associated with survival and response in HNSCC patients receiving ICIs

To account for correlations among the various peripheral blood biomarkers examined, we performed multivariable analysis using the elastic net variable selection method. Refitted multivariable models adjusted for ECOG PS, p16 status, and smoking confirmed that lower % lymphocytes and higher LDH and absolute neutrophils correlated with worse OS and PFS (Tables 6 and 7). Lower % lymphocytes and higher LDH also correlated with worse ORR (Table 8).

**TABLE 6:** Multivariable model for factors associated with OS.

	OS		
	HR	95% CI	*p*‐value
ECOG PS	1.84	1.37–2.46	<0.001
p16 positive versus negative	0.95	0.62–1.46	0.825
Former versus current smoker	1.17	0.66–2.08	0.597
Never versus current smoker	1.04	0.58–1.88	0.893
Lymphocytes (%)^a^	0.18	0.07–0.47	<0.001
LDH^a^	3.89	1.55‐9.79	0.004
Neutrophils (abx)^a^	1.45	0.44–4.75	0.538

^a^ Log10 transformed.

**TABLE 7:** Multivariable model for factors associated with PFS.

	PFS		
	HR	95% CI	*p*‐value
ECOG PS	1.60	1.20–2.12	0.001
p16 positive versus negative	1.07	0.71–1.59	0.750
Former versus current smoker	1.08	0.63–1.87	0.777
Never versus current smoker	0.98	0.57–1.69	0.945
Lymphocytes (%)^a^	0.33	0.13–0.82	0.017
LDH	2.92	1.23‐6.94	0.015
Neutrophils (abx)^a^	2.32	0.74–7.23	0.148

^a^ Log10 transformed.

**TABLE 8:** Multivariable model for factors associated with ORR.

	Objective response		
	OR	95% CI	*p*‐value
ECOG PS	0.37	0.16–0.79	<0.001
p16 positive versus negative	2.00	0.81–4.92	0.129
Former versus current smoker	0.30	0.09–1.00	0.049
Never versus current smoker	0.29	0.09–0.96	0.043
Lymphocytes (%)^a^	7.22	0.98–63.46	0.061
LDH^a^	0.04	0.00‐0.64	0.048

^a^ Log10 transformed.

## 4 DISCUSSION

In the largest cohort to date to examine PBBMs in R/M HNSCCs treated with ICIs, we identified PBBMs associated with survival and response using elastic net variable selection. While this represents one of the first studies of PBBMs in the immunotherapy population for head and neck cancer, PBBMs have been previously studied in general prognostication for head and neck cancer.

Numerous mechanisms link carcinogenesis and inflammation in a reciprocal way, as chronic inflammation promotes carcinogenesis while intratumoral inflammatory cells produce inflammatory mediators leading to tumor progression and metastasis.^14^ In various solid tumors, including head and neck cancer, both tumor cells and tumor microenvironment have been shown to stimulate a systemic inflammatory response, leading to increased peripheral platelets, neutrophils, and monocytes.^15^, ^16^, ^17^ In the tumor microenvironment, neutrophils secrete proteases, cytokines, and growth factors, including interleukin (IL)‐6, IL‐8, epidermal growth factor and vascular endothelial growth factor, that lead to tumor cell activation, invasion, and metastasis.^18^ In contrast, decreased local lymphocytes cause decreased production of cytokines that promote apoptosis of tumor cells, leading to attenuation of monitoring by the host immune response.^19^, ^20^, ^21^ Being able to sample the peripheral blood as a correlate of the tumor microenvironment is an attractive avenue for biomarker development due to the low cost, ease of sampling, and existing infrastructure to compare results across laboratories.

Several studies in HNSCC, which have examined a variety of treatment modalities and anatomic subsites, have generally found that higher peripheral neutrophil‐to‐lymphocyte ratio (NLR), higher neutrophils and monocytes, higher PNI, and lower lymphocytes are associated with worse survival.^13^, ^15^ We report consistent findings in the immunotherapy population for R/M HNSCC, which has not previously been so comprehensively studied with mature follow‐up times due to the relatively recent emergence of ICIs for this disease. Our robust multivariable analysis, which accounted for correlations among the individual peripheral blood parameters using the elastic net variable selection method, revealed PBBMs (% lymphocytes and LDH) associated with ORR as well as survival. In a study of 34 R/M HNSCC, Ho et al. found that lower pretreatment lymphocyte count was associated with response to PD‐1 inhibitors.^22^ Our study identifies additional PBBMs associated with response and corroborates prior literature to highlight the importance of pretreatment lymphocytes in prognosticating response. Our observation that lower baseline % lymphocytes correlate with worse OS, PFS, and ORR may reflect tumor resistance to ICI therapy in particular, as ICIs rely on reactivation of lymphocytes to exert their anti‐tumor effects.

Furthermore, our study showed for the first time that pretreatment elevated serum LDH is a poor prognostic marker for OS, PFS, and ORR to immunotherapy in R/M HNSCC. Serum LDH has been well‐studied as an important biomarker in certain solid tumors, most notably in non‐Hodgkin's lymphoma and metastatic melanoma, where it is used in staging.^23^, ^24^ More limited studies have found LDH to have prognostic significance in head and neck cancers as well.^25^, ^26^, ^27^, ^28^ LDH is a key enzyme in anaerobic glycolysis through conversion of pyruvate to lactate and is thought to reflect more aggressive cancers, which are able to proliferate under hypoxic conditions.^25^, ^29^ Hypoxic tumor conditions are well‐known to lead to resistance to radiation and chemotherapy, indicating that elevated LDH may be an indirect marker of resistance to these treatment modalities.^30^ Our novel findings suggest that elevated serum LDH may also represent resistance to ICIs as well.

Although our study did not demonstrate any PBBMs that correlated with serious adverse events, we did not specifically examine immune‐related adverse events (irAEs). These irAEs are auto‐immune toxicities that can affect any organ system and are uniquely related to treatment with ICIs compared to standard chemotherapy. They are known to have a delayed onset and longer duration than standard chemotherapy‐related toxicities.^23^ Our toxicity analysis window of 100 days of treatment may not have adequately captured irAEs, and separate examination of PBBMs in relation to irAEs in needed.

Our work has several limitations, including the retrospective design, inclusion of non‐FDA approved PD‐1 inhibitor treatment regimens (although this cohort was the minority), as well as heterogeneity of the patient population which included smaller groups of less prevalent primary sites such as skin, paranasal sinus, and auditory canal. Additionally, as most patients included in this study began ICI treatment before the routine use of the CPS biomarker, not enough data existed to allow correlation of our findings with the validated CPS biomarker.

Our work demonstrates strong evidence for prognostic PBBMs, and ongoing work from our group aims to develop predictive PBBM models that can be readily applied in the clinical setting for improved patient selection and counseling for ICI treatment.

## 5 CONCLUSIONS

In the largest study to date to examine PBBMs in R/M HNSCCs treated with ICIs, our variable selection method showed that baseline lower % lymphocytes as well as higher LDH and absolute neutrophils correlated with worse OS and PFS, and lower % lymphocytes and higher LDH correlated with worse ORR. PBBMs are promising prognostic tools for immunotherapy in HNSCC and warrant further investigation in a large, prospective study along with validation with CPS biomarker.

## ETHICAL APPROVAL STATEMENT

This study was reviewed and approved by the Fred Hutchinson Cancer Research Institutional Review Board.

## AUTHOR CONTRIBUTIONS

**Cassie Pan:** Conceptualization (equal); data curation (equal); funding acquisition (supporting); investigation (equal); visualization (lead); writing – original draft (lead). **Vicky Wu:** Conceptualization (equal); formal analysis (equal); methodology (equal); software (equal); writing – review and editing (equal). **Jenna Voutsinas:** Conceptualization (equal); formal analysis (equal); methodology (equal); software (equal); writing – review and editing (equal). **Jeffrey J Houlton:** Resources (equal); supervision (supporting); writing – review and editing (equal). **Brittany Barber:** Resources (equal); writing – review and editing (equal). **Zain H Rizvi:** Resources (equal); writing – review and editing (equal). **Emily Marchiano:** Resources (equal); writing – review and editing (equal). **Neal Futran:** Resources (equal); writing – review and editing (equal). **George E. Laramore:** Resources (equal); writing – review and editing (equal). **Jay Liao:** Resources (equal); writing – review and editing (equal). **Upendra Parvathaneni:** Resources (equal); writing – review and editing (equal). **Renato G Martins:** Resources (equal); writing – review and editing (equal). **Jonathan R Fromm:** Resources (equal); writing – review and editing (equal). **Cristina P. Rodriguez:** Conceptualization (equal); funding acquisition (lead); investigation (equal); methodology (equal); resources (equal); supervision (lead); writing – review and editing (equal).

## FUNDING INFORMATION

National Institutes of Health T32 DC000018 (Dr. Pan).

## CONFLICT OF INTEREST STATEMENT

Dr. Futran reported educational consultancy role for Stryker Corporation. Dr. Rodriguez reported receipt of institutional research funding from AstraZeneca, Ayala, Bristol Myers Squibb, Ignyta, and Merck, and reported advisory board membership for Cue Biopharma. The other authors declare no potential conflicts of interest.

## Data Availability

The data that support the findings of this study are available from the corresponding author upon reasonable request.

## References

[1] Cohen EEW , Bell RB , Bifulco CB , et al. The Society for Immunotherapy of Cancer consensus statement on immunotherapy for the treatment of squamous cell carcinoma of the head and neck (HNSCC). J Immunother Cancer. 2019;7:184. doi:10.1186/s40425-019-0662-5 31307547 PMC6632213

[2] Cohen EEW , Soulières D , Le Tourneau C , et al. Pembrolizumab versus methotrexate, docetaxel, or cetuximab for recurrent or metastatic head‐and‐neck squamous cell carcinoma (KEYNOTE‐040): a randomised, open‐label, phase 3 study. Lancet. 2019;393(10167):156‐167. doi:10.1016/S0140-6736(18)31999-8 30509740

[3] Rischin D , Harrington KJ , Greil R , et al. Protocol‐specified final analysis of the phase 3 KEYNOTE‐048 trial of pembrolizumab (pembro) as first‐line therapy for recurrent/metastatic head and neck squamous cell carcinoma (R/M HNSCC). JCO. 2019;37(15_suppl):6000. doi:10.1200/JCO.2019.37.15_suppl.6000

[4] Ferris RL , Blumenschein G , Fayette J , et al. Nivolumab for recurrent squamous‐cell carcinoma of the head and neck. N Engl J Med. 2016;375(19):1856‐1867. doi:10.1056/NEJMoa1602252 27718784 PMC5564292

[5] Burtness B , Harrington KJ , Greil R , et al. Pembrolizumab alone or with chemotherapy versus cetuximab with chemotherapy for recurrent or metastatic squamous cell carcinoma of the head and neck (KEYNOTE‐048): a randomised, open‐label, phase 3 study. Lancet. 2019;394(10212):1915‐1928. doi:10.1016/S0140-6736(19)32591-7 31679945

[6] Pan C , Wu Q (Vicky) , Voutsinas J , et al. Neutrophil to lymphocyte ratio and peripheral blood biomarkers correlate with outcomes among patients with recurrent metastatic head and neck squamous cell carcinoma and salivary gland cancer treated on a phase II trial of pembrolizumab and vorinostat. In: American Head and Neck Society 2022 Annual Meeting at COSM. AHNS; Abstract nr 119950; 2022.

[7] Foster CC , Kochanny S , Khattri A , et al. Association of a baseline neutrophil‐to‐lymphocyte ratio (NLR) with progression‐free and overall survival in head and neck cancer patients receiving anti‐PD‐1 therapy. JCO. 2018;36(15):6038. doi:10.1200/JCO.2018.36.15_suppl.6038

[8] Nishikawa D , Suzuki H , Koide Y , et al. Prognostic markers in head and neck cancer patients treated with nivolumab. Cancers (Basel). 2018;10(12):E466. doi:10.3390/cancers10120466 PMC6316608 30477171

[9] Hong S , Zhou T , Fang W , et al. The prognostic nutritional index (PNI) predicts overall survival of small‐cell lung cancer patients. Tumor Biol. 2015;36(5):3389‐3397. doi:10.1007/s13277-014-2973-y 25527156

[10] Lee JY , Kim HI , Kim YN , et al. Clinical significance of the prognostic nutritional index for predicting short‐ and long‐term surgical outcomes after gastrectomy: a retrospective analysis of 7781 gastric cancer patients. Medicine. 2016;95(18):e3539. doi:10.1097/MD.0000000000003539 27149460 PMC4863777

[11] Minami S , Ogata Y , Ihara S , Yamamoto S , Komuta K . Pretreatment Glasgow prognostic score and prognostic nutritional index predict overall survival of patients with advanced small cell lung cancer. LCTT. 2017;8:249‐257. doi:10.2147/LCTT.S142880 PMC5726358 29263709

[12] Johannet P , Sawyers A , Qian Y , et al. Baseline prognostic nutritional index and changes in pretreatment body mass index associate with immunotherapy response in patients with advanced cancer. J Immunother Cancer. 2020;8(2):e001674. doi:10.1136/jitc-2020-001674 33219093 PMC7682457

[13] Luan CW , Tsai YT , Yang HY , Chen KY , Chen PH , Chou HH . Pretreatment prognostic nutritional index as a prognostic marker in head and neck cancer: a systematic review and meta‐analysis. Sci Rep. 2021;11(1):17117. doi:10.1038/s41598-021-96598-9 34429476 PMC8385102

[14] Colotta F , Allavena P , Sica A , Garlanda C , Mantovani A . Cancer‐related inflammation, the seventh hallmark of cancer: links to genetic instability. Carcinogenesis. 2009;30(7):1073‐1081. doi:10.1093/carcin/bgp127 19468060

[15] Valero C , Pardo L , López M , et al. Pretreatment count of peripheral neutrophils, monocytes, and lymphocytes as independent prognostic factor in patients with head and neck cancer. Head Neck. 2017;39(2):219‐226. doi:10.1002/hed.24561 27534525

[16] Khunger M , Patil PD , Khunger A , et al. Post‐treatment changes in hematological parameters predict response to nivolumab monotherapy in non‐small cell lung cancer patients. PLoS One. 2018;13(10):e0197743. doi:10.1371/journal.pone.0197743 30359383 PMC6201866

[17] Dirican N , Karakaya YA , Gunes S , Daloglu FT , Dirican A . Association of intra‐tumoral tumour‐infiltrating lymphocytes and neutrophil‐to‐lymphocyte ratio is an independent prognostic factor in non‐small cell lung cancer. Clin Respir J. 2017;11(6):789‐796. doi:10.1111/crj.12417 26619201

[18] Hanahan D , Weinberg RA . Hallmarks of cancer: the next generation. Cell. 2011;144(5):646‐674. doi:10.1016/j.cell.2011.02.013 21376230

[19] Yu Y , Wang H , Yan A , et al. Pretreatment neutrophil to lymphocyte ratio in determining the prognosis of head and neck cancer: a meta‐analysis. BMC Cancer. 2018;18:383. doi:10.1186/s12885-018-4230-z 29618336 PMC5885417

[20] Hanahan D , Coussens LM . Accessories to the crime: functions of cells recruited to the tumor microenvironment. Cancer Cell. 2012;21(3):309‐322. doi:10.1016/j.ccr.2012.02.022 22439926

[21] Schreiber RD , Old LJ , Smyth MJ . Cancer immunoediting: integrating immunity's roles in cancer suppression and promotion. Science. 2011;331(6024):1565‐1570. doi:10.1126/science.1203486 21436444

[22] Ho WJ , Yarchoan M , Hopkins A , Mehra R , Grossman S , Kang H . Association between pretreatment lymphocyte count and response to PD1 inhibitors in head and neck squamous cell carcinomas. J Immunother Cancer. 2018;6(1):84. doi:10.1186/s40425-018-0395-x 30170629 PMC6117944

[23] International Non‐Hodgkin's Lymphoma Prognostic Factors Project . A predictive model for aggressive non‐Hodgkin's lymphoma. N Engl J Med. 1993;329(14):987‐994. doi:10.1056/NEJM199309303291402 8141877

[24] Nikolin B , Djan I , Trifunovic J , et al. MIA, S100 and LDH as important predictors of overall survival of patients with stage IIb and IIc melanoma. J BUON. 2016;21(3):691‐697. 27569092

[25] Uehara T , Doi H , Ishikawa K , et al. Serum lactate dehydrogenase is a predictive biomarker in patients with oropharyngeal cancer undergoing radiotherapy: retrospective study on predictive factors. Head Neck. 2021;43(10):3132‐3141. doi:10.1002/hed.26814 34268826 PMC8457164

[26] Wan X , Wei L , Li H , et al. High pretreatment serum lactate dehydrogenase level correlates with disease relapse and predicts an inferior outcome in locally advanced nasopharyngeal carcinoma. Eur J Cancer. 2013;49(10):2356‐2364. doi:10.1016/j.ejca.2013.03.008 23541571

[27] Guo E , Guo L , An C , et al. Prognostic significance of lactate dehydrogenase in patients undergoing surgical resection for laryngeal squamous cell carcinoma. Cancer Control. 2020;27(1):1073274820978795. doi:10.1177/1073274820978795 33297727 PMC8480349

[28] Ban EJ , Kim D , Kim JK , et al. Lactate dehydrogenase a as a potential new biomarker for thyroid cancer. Endocrinol Metab (Seoul). 2021;36(1):96‐105. doi:10.3803/EnM.2020.819 33677931 PMC7937852

[29] Höckel M , Vaupel P . Tumor hypoxia: definitions and current clinical, biologic, and molecular aspects. J Natl Cancer Inst. 2001;93(4):266‐276. doi:10.1093/jnci/93.4.266 11181773

[30] Muz B , de la Puente P , Azab F , Azab AK . The role of hypoxia in cancer progression, angiogenesis, metastasis, and resistance to therapy. Hypoxia (Auckl). 2015;3:83‐92. doi:10.2147/HP.S93413 27774485 PMC5045092

